# A Case of Labour, Attended by Some Unusual Appearances

**Published:** 1842-01

**Authors:** John Overton

**Affiliations:** Nashville, Tenn.


**Art. II.**—*A case of Labour attended by some unusual appearances.* By John Overton, M. D., of Nashville, Tenn.

Mary, a coloured woman, was seized with labour-pains, and all the concomitant symptoms of child bearing on the —— day of —— 1822. The pains were importunate and severe, accompanied by much restlessness and sudden, often-repeated and rude tossing of the body in different directions. The woman was ungovernable by the midwife, who was in attendance, and in despite of every admonition to the contrary, continued to throw herself, with considerable violence, alternately on her back and abdomen upon which she ultimately fell upon a bench. At eleven o'clock at night, succeeding the time of its commencement, the labour subsided, immediately after much tossing and agitation of the body, the fall upon the bench, before noticed.

The child, which, anterior to this occurrence, descended perceptibility into the superior strait of the pelvis, now suddenly receded and was no longer perceptible to the touch, per vias naturales. The abdomen, which, before, was tense and apparently collected about the uterine region, was now relaxed; without any contraction of its muscular portions, and diffused equally over this region, to a much greater extent than is usual in parturient women; or even in the latter months of gestation. Until this period the labour had been marked by strong and frequently repeated bearing-down pains, together with every other exertion of muscularity usually and necessarily attendant on parturition. These, however, were suddenly subsided, and, in lieu thereof, the woman ceased to toss about, remained still, on her back, and complained of a heavy pain constantly present about the umbilicus, and expressed her sensations of bodily suffering by grunts, frequently repeated, and at regular intervals; but without referring their production to any cause, necessarily in connection with the parturient state. She felt pain about the umbilicus; was sick at the stomach, and occasionally vomited, and felt general disquietude and uneasiness of body; but nothing like an effort of the womb and abdominal parietes, to finish a process which had been commenced with such pressing activity. The countenance was *wild* and affrighted, and seemed indicative of a mind despondent in its hopes, defeated in its anticipations, and bowed down under the weight of a calamity which no art could evade or strength overcome. The arterial system, however, did not participate in the state of the mind or body. The pulse was sufficiently full, somewhat accelerated beyond what is usual in parturient women, but was moderately soft, round, regular, open, and slow, as respects the quickening of its pulsations. Judging by the pulse alone, there appeared nothing in the case to excite alarm; or which demanded the intervention of art, in the consummation of the delivery. The os tincæ, and other structures mechanically operative or passive in the process of child-bearing, were in a state of entire relaxation and the fœtus having receded from the superior strait of the pelvis rested upon, and by a considerable part of its mass, hung over the pelvis upon the abdominal muscles. Such was the condition of this woman when I first visited her, which was twenty-six hours after the distinct existence of labour, and fifteen after the cessation of all the uterine exertions.

Reflecting upon the phenomena furnished by the case, I was enabled, in a short time, to settle in my own mind its pathological character, as well as the indications, which it was indispensable to accomplish, to obtain relief. I concluded that the abdominal muscles had *certainly*, and the womb *probably*, suffered a mechanical injury in consequence of the excessive tossing and tumbling, and fall of the woman in the first stage of the labour; and that the cessation of the parturient exertions, being the result of a cause so durable in its nature as a bruise or laceration, were not likely again to recur; in no event soon enough to satisfy the present necessities of the system. Under these presentations I determined to turn and deliver the fœtus by the feet, without further attendance upon the efforts of nature to furnish any assistance, which she had already obstinately denied by fifteen hours of delay. The feet were consequently brought into the vagina, yet still indulging a feeble hope that nature *might* possibly resume her work, under circumstances promising a more speedy and felicitious result, they were permitted to remain in this situation for the space of two hours, awaiting the renewal of the actions of the uterus to complete the delivery. Uterine action, however, did not occur, and the child was delivered by the efforts of the accoucheur alone, without any perceptible effort of the womb, or abdominal muscles promotive of this result; which was produced, not without much more assiduity and circumspection than is necessary in the management of ordinary births when effected by turning. The expulsive efforts of the womb having entirely ceased, there existed no power independent of the accoucheur which was at all calculated to establish the necessary relationship between the head of the fœtus and the varying dimensions of the outlet through which it was to pass.

Correctly to effect a proper adaptation of the short diameter of the head to the largest diameter of the pelvis, as varied by different portions of its extent, retarded the delivery, and constituted, after the turning was accomplished, the chief difficulty with which the accoucheur had to combat.

The fœtus having been delivered, was separated from the placental attachment, and the woman put to bed; still hoping that the womb might, after so long a respite from exertion, resume its activity, and expel the placenta from its attachments. For this purpose the patient remained without any interference, save that of greatly agitating the abdominal parietes over the uterine region, for four hours without the least appearance of contraction in the womb, or abdominal muscles. The abdomen remained soft, and as large as before delivery; manifesting an entire insensibility to the impression of any irritation to which it was subjected.

At this time the occurrence of profuse hemorrhage induced me to deliver the placenta with as much expedition as practicable. It was found in situ naturale, and detached from the womb but by a small portion of its circumference; and was removed by means as independent of uterine exertion, as those which before had relieved this viscus from the burden of the fœtus.

After the delivery of the placenta the hemorrhage soon ceased, and the lochial evacuation which succeeded was unattended with any character in respect either to extent or duration, which rendered it peculiar; and except the relaxed and protuberant state of the abdomen, and an inconsiderable sensation of pain attributed to the region of the umbilicus, all the phenomena now portended a favorable recovery. In this condition the patient was left, with general direction for her treatment during her confinement, and a request, that should any unusual symptom occur, to send me word of it.

After a few days I was requested to visit this woman again, and found her labouring under most of the symptoms which are usually the result of mechanical violence done to any important viscus; with the modifications of these symptoms, which is perhaps always attendant upon serious lesions of the womb. The prominent causes of complaint, were a hard and painful tumor, phlegmonous in its aspect, of a circular form, of about four or five inches in diameter, the umbilicus occupying about the centre of the diseased circle. The tumor was but slightly elevated above the healthy surface of the abdomen, but in all other respects very accurately resembled an ordinary phlegmon of the abdominal parietes.

Believing that these appearances were to be attributed to mechanical injury done to the womb and abdominal muscles at the time of delivery, I directed an abstemious diet, small portions of antimony as a febrifuge, and a linseed poultice applied warm and kept constantly over the whole surface of the tumor.

This treatment was continued with but little variation, until free suppuration had been established; which was indicative as in other phlegmonious tumors, by pointing towards its centre, and an evident fluctuation of its contents from percussion with the fingers. The tumor was now opened in the ordinary manner with a lancet, and discharged at the time about half a pint of matter greatly resembling ordinary pus, though somewhat more thin and gluey in its appearance. From this time the tumor and all the symptomatic effects which had attended its progress gradually declined; and after the lapse of five or six weeks the discharge entirely ceased and the wound was entirely cicatrised.

I have seen this woman within a few weeks. She now enjoys fine health, has grown corpulent, but has never been pregnant sice the accident above described; although at the time of its occurrence she was not more than 18 years of age. Since the accident she has menstruated.

It may be proper to add, that, at the time of delivery, there was no evidence of any injury done to the abdominal parietes, competent to account for the sudden suspension of uterine action, which occurred during the progress of the labour. There was no wound or perceptible bruise of the abdomen, or complaint of pain from injury done to this surface. I came, therefore, to the conclusion, at the time, that the womb had beeen ruptured: although the abdominal covering had suffered little comparative injury. The probable condition of the different parts subjected to the action of the same lædent cause enables me to confide in the truth of this conclusion; the abdomen was comparatively relaxed, the womb in a state of strong contraction at the time of the fall and hence the dissimilarity of its effect upon the two parts submitted to its influence.

Nashville, Sept. 8, 1841

